# MathOdyssey: Benchmarking Mathematical Problem-Solving Skills in Large Language Models Using Odyssey Math Data

**DOI:** 10.1038/s41597-025-05283-3

**Published:** 2025-08-08

**Authors:** Meng Fang, Xiangpeng Wan, Fei Lu, Fei Xing, Kai Zou

**Affiliations:** 1 Department of Computer Science, University of Liverpool, Liverpool, UK; 2 NetMind.AI, London, UK; 3 Department of Mathematics, Johns Hopkins University, Baltimore, MD USA; 4 Mathematica Policy Research, Princeton, New Jersey USA

**Keywords:** Scientific data, Computer science

## Abstract

Large language models (LLMs) have significantly advanced natural language understanding and demonstrated strong problem-solving abilities. Despite these successes, most LLMs still struggle with solving mathematical problems due to the intricate reasoning required. To support rigorous evaluation of mathematical reasoning in LLMs, we introduce the “MathOdyssey” dataset - a curated collection of 387 expert-generated mathematical problems spanning high school, university, and Olympiad-level topics. Each problem is accompanied by a detailed solution and categorized by difficulty level, subject area, and answer type. The dataset was developed through a rigorous multi-stage process involving contributions from subject experts, peer review, and standardized formatting. We provide detailed metadata and a standardized schema to facilitate consistent use in downstream applications. To demonstrate the dataset’s utility, we evaluate several representative LLMs and report their performance across problem types. We release MathOdyssey as an open-access resource to enable reproducible and fine-grained assessment of mathematical capabilities in LLMs and to foster further research in mathematical reasoning and education.

## Background & Summary

Large language models (LLMs) have demonstrated exceptional proficiency in mastering human language and handling mathematical problems, including typical routine math problems^1–3^. In recent years, several benchmarks related to mathematics have been proposed, such as the GSM8K dataset^4^, the MATH dataset^5^ and so on. Recent LLMs and reasoning approaches have addressed these problems with notable success^1–3,6–8^. For instance, GPT-4, using advanced prompting techniques^1^, has achieved more than a 90% success rate on GSM8K and 80% on MATH. These achievements indicate that LLMs possess remarkable capabilities in mathematical reasoning.

The quest to improve LLMs’ mathematical problem-solving abilities is not just a demonstration of technological advancement but a crucial step toward developing more general and capable artificial intelligence systems^9^. On the one hand, this endeavor requires datasets that accurately measure and challenge the AI’s mathematical reasoning beyond basic problems^8,10^. Although their performance is high on datasets like GSM8K^4^, it remains uncertain how well they handle more complex mathematical challenges, such as those found in university-level courses and competitive high school mathematics. Performance may diminish significantly in these areas. This gap highlights the ongoing need for enhanced mathematical reasoning capabilities in AI, a critical area for assessing cognitive abilities akin to human intelligence. Moreover, a significant obstacle is that many existing datasets might have been included in the training phases of these models, potentially skewing performance metrics. Prominent examples include STEM-Q^11^, GSM8K^4^, and the MATH dataset^5^, which may no longer provide a true test of an LLM’s mathematical capabilities. On the other hand, high-quality, expert-crafted original problems are scarce. For instance, a study testing GPT-4^12^ included only 105 such problems in high school and university-level science and math.

To directly address these challenges, we introduce the “MathOdyssey” dataset, a rigorously curated collection of 387 mathematical problems for evaluating the general mathematical capacities of LLMs. See examples in Table 1. The MathOdyssey dataset features a spectrum of questions from Olympiad-level competitions, advanced high school curricula, and university-level mathematics. All problems were created by mathematics professionals, including high-school educators, researchers, and university professors. The key distinction of our dataset is its expert-driven creation, which minimizes the risk of data contamination.

**Table 1:** MathOdyssey dataset examples.

**Olympiad-level**
**Problem:** Let $S=\left\{1,2,\cdots 2024\right\}$, if the set of any *n* pairwise prime numbers in *S* has at least one prime number, the minimum value of *n* is —. **Answer:** 16. **Reasoning:** Taking the 15 numbers 1, 2^2^, 3^2^, … , 43^2^. They violate the condition. Furthermore, since S does not contain any non-prime numbers with a minimum prime factor of at least 47 (because 47^2^ > 2024). Set 1 aside, there are only 14 types of non-prime numbers in S, classified by its minimum prime factor. Applying the Pigeonhole Principle, we conclude that n = 16.
**High School** **Problem:** What are the solutions of the quadratic equation 15*x*^2^ = 2*x* + 8. $\left.{\rm{A}}\right)\,\left\{-\frac{4}{3},-\frac{3}{2}\right\}\,\,\left.{\rm{B}}\right)\,\left\{-\frac{4}{5},\frac{2}{3}\right\}\,\,\left.{\rm{C}}\right)\,\left\{-\frac{3}{2},\frac{4}{5}\right\}\,\,\left.{\rm{D}}\right)\,\left\{-\frac{2}{3},\frac{4}{5}\right\}$ **Answer:** *D* **Reasoning:** First move all terms to one side: 15*x*^2^ − 2*x* − 8 = 0. Then factor into (5*x* − 4)(3*x* + 2) = 0. Setting 5*x* − 4 to zero results in a solution of $x=\frac{4}{5}$ and setting 3*x* + 2 to zero results in a solution of $x=-\frac{2}{3}$.
**University-level** **Problem:** Find the limit $\mathop{\mathrm{lim}}\limits_{x\to 1}\frac{f(2{x}^{2}+x-3)-f(0)}{x-1}$ given $f{\prime} (1)=2$ and $f{\prime} (0)=-\,1$. **Answer:** −5. **Reasoning:** Let *g*(*x*) = 2*x*^2^ + *x* − 3. Since *g*(1) = 0, the desired limit equals $\mathop{\mathrm{lim}}\limits_{x\to 1}\frac{f(g(x))-f(g(1))}{x-1}$. By the definition of the derivative and the chain rule and noting that ${g}^{{\prime} }(1)=5$, we have $\mathop{\mathrm{lim}}\limits_{x\to 1}\frac{f(g(x))-f(g(1))}{x-1}=f{\prime} (g(1))g{\prime} (1)=f{\prime} (0)g{\prime} (1)=(-1)(5)=-\,5.$

We demonstrate three distinct levels to challenge various aspects of mathematical knowledge: Olympiad-level, High School, and University-level mathematics. Each example consists of three parts: the problem, the answer, and the reasoning.

Furthermore, we show the process of dataset construction, validation, and annotation. We provide detailed statistics on problem difficulty, subject distribution, and answer types. To illustrate the dataset’s use, we include a comparative analysis of LLM performance across categories, highlighting its potential to support research in mathematical AI.

The MathOdyssey dataset is released as an open resource to enable reproducible evaluation and support future work in AI reasoning. Our contributions are as follows:

- We introduce a new mathematical dataset that provides different levels of mathematical problems and covers a wider range of subject areas.
- We open source the MathOdyssey benchmark dataset, a meticulously curated collection of mathematical problems spanning various domains and levels, complete with natural language solutions. This dataset is specifically designed to probe the reasoning abilities of LLMs, offering a unique tool for assessing AI performance in complex mathematical reasoning. Each question has an objective answer serving as -ground-truth’, allowing for objective evaluation on the LLM outputs. In particular, the Open-Answer problems emphasize the importance of detailed reasoning and solution.
- We conduct a comprehensive benchmark analysis using our dataset on both open-source and closed-source LLMs. Our findings reveal that while closed-source models currently lead, open-source models are rapidly catching up, highlighting the competitive landscape of LLM capabilities in mathematical problem-solving.

## Related Work

### Large Language Models for Mathematics

Applying large language models (LLMs) to mathematical problems has led to significant strides, though solving such problems remains challenging due to the need for highly complex and symbolic multi-step reasoning capabilities. Both GPT-3.5 and GPT-4^1^ have shown promising reasoning abilities for complex mathematical tasks, such as those in the MATH dataset^5^. However, the performance of open-source models, like Llama-1 and Llama-2^2^, is still far from satisfactory in this domain. To enhance the mathematical problem-solving abilities of LLMs, prompt-based methods have also been developed^6,13,14^. These methods aim to improve reasoning and accuracy by guiding the models through structured prompts that help in breaking down complex problems into manageable steps.

### Mathematical Evaluation for Large Language Models

Evaluating the mathematical capacity of large language models (LLMs) is crucial. Benchmarks such as GSM8K^4^, which targets middle-school level mathematics, and MATH^5^, which focuses on high-school math competitions, have been widely used. For university-level problems, datasets like ProofNet^15^ and OCWCourses^16^ are prominent. Additionally, MiniF2F^17^ and AlphaGeometry^8^ provide Olympiad-level problems, while the SAT dataset^18^ includes problems from the College Board SAT examination. These datasets have limitations, particularly at the undergraduate level and above, where they fall short in addressing graduate-level and competition-level difficulties^10^. To address this gap, we introduce the MathOdyssey dataset, a diverse collection of mathematical problems designed to serve as a rigorous benchmark for assessing both open-source and closed-source models. Table 2 highlights the properties of MathOdyssey compared to relevant benchmarks, emphasizing the different levels and the diversity of subject areas and question types in our benchmark. This dataset spans a spectrum of difficulty levels, from high school to advanced university mathematics, highlighting the evolving capabilities and ongoing challenges in LLM mathematical problem-solving.

**Table 2:** Comparison of existing evaluation datasets for testing AI in mathematics.

Dataset	Year	Description	# of Test
GSM8k^4^	2021	8.5k middle-school level math word problems	1k
MATH^5^	2021	12.5k high-school math competitions	5k
OCWCourses^16^	2022	University-level, MIT’s OpenCourseWare	272
MiniF2F^17^	2023	Olympiad-level	488
SAT^18^	2023	Figureless questions from SAT	32
ProofNet^15^	2023	University-level, proofs	371
AlphaGeometry^8^	2024	Olympiad Geometry only	30
MathOdyssey (this work)	2024	High School, University-level, Olympiad-level	387

These datasets are limited, especially in the availability of high-quality, expert-crafted original problems with varying difficulty levels.

## Methods

The MathOdyssey dataset was meticulously designed to evaluate the mathematical reasoning capabilities of large language models (LLMs). The creation process involved structured stages, including expert recruitment, problem development, review, formatting, and categorization. This section outlines each stage to ensure transparency and reproducibility.

### Expert Recruitment

To ensure the quality, originality, and academic rigor of the dataset, contributors were recruited through direct invitation by the AGI Odyssey executive committee as part of the Global Artificial Intelligence Championships (GAIC) Math 2024 initiative. All contributors were mathematics professionals with demonstrated expertise in teaching, research, or competition-level problem design.

The cohort included:

- Professors from universities such as Arizona State University, Drexel University, etc;
- High school educators experienced in mathematics instruction and Olympiad coaching;
- Applied mathematics researchers and specialists in symbolic reasoning.

Selection criteria emphasized disciplinary expertise, teaching experience at the relevant educational levels, and prior involvement in assessment or curriculum development. All contributors provided informed consent to participate in the dataset creation process under an approved research protocol.

### Problem Design and Annotation

Contributors were tasked with creating original mathematical problems across three difficulty levels-High School, University, and Olympiad-level. Each problem was written in LaTeX and accompanied by:

- A canonical solution (final answer),
- A detailed reasoning annotation that outlines a step-by-step explanation of the solution,
- Metadata indicating the problem’s difficulty level, subject area, and answer format.

Problems were designed to cover a wide range of mathematical domains, including Algebra, Geometry, Number Theory, Combinatorics, Calculus, Linear Algebra, Differential Equations, Probability, and Statistics. The inclusion of structured reasoning enables future work in explainability and chain-of-thought supervision. All problems were authored independently and were not drawn from or adapted from existing datasets, reducing the risk of training contamination.

### Problem Categorization and Structure

Each problem in the MathOdyssey dataset is annotated with structured metadata to support rigorous evaluation and analysis. The core metadata fields include:

- **Difficulty Level**:
  - *High School*: Foundational topics such as geometry, algebra, and pre-calculus.
  - *University*: Undergraduate-level topics such as calculus, linear algebra, probability, statistics, and differential equations.
  - *Olympiad*: Competition-style problems requiring deep reasoning and advanced mathematical insight.
- **Answer Type**:
  - *True/False (T-F)*: Assessing the correctness of a given mathematical statement.
  - *Multiple Choice (MCQ)*: Selecting the correct answer from a list of options.
  - *Open-Answer (Open)*: Producing a solution in the form of a real number, symbolic expression, vector, or matrix.
- **Reasoning Annotation**: In addition to the final answer, each problem is accompanied by a detailed, step-by-step solution. This reasoning is written in natural mathematical language (in LaTeX format) and reflects the canonical method used by expert contributors. These annotations are essential for understanding problem difficulty, training interpretable models, and enabling explainable evaluation pipelines.

This structured approach ensures the dataset can support a variety of downstream tasks, from automated evaluation to few-shot learning and reasoning chain supervision.

### Independent Review and Validation

Following problem creation, each question underwent a multi-stage validation process overseen by the AGI Odyssey executive committee. An independent reviewer-also a mathematics expert-was assigned to evaluate each problem for correctness, clarity, originality, and alignment with its labeled difficulty and subject area.

Reviewers flagged ambiguities, corrected errors, and suggested refinements where appropriate. Authors were then asked to revise problems based on this feedback. The executive committee coordinated this review workflow and maintained final editorial oversight. All 387 problems in MathOdyssey passed through this pipeline before inclusion in the dataset.

This structured review and revision cycle ensured that each problem meets a high standard of rigor, educational value, and clarity. Problems were also tested for automatic answer validation across all three answer types (True/False, Multiple Choice, Open-ended), enabling consistent and reproducible evaluation.

### Data Formatting and Delivery

All problems and solutions were formatted consistently to ensure accessibility and usability. Each item was stored in a structured JSON format with fields for the problem statement, solution, reasoning, difficulty level, subject area, and answer type. Content is also available in LaTeX and PDF formats to support various downstream use cases.

## Data Record

The MathOdyssey dataset^19^ is publicly available to support research in mathematical reasoning and large language model (LLM) evaluation, and can be accessed at 10.5281/zenodo.15298048. It includes a structured collection of 387 original mathematical problems, accompanied by rich metadata, solutions, and supporting software. The dataset is hosted on GitHub and Hugging Face, with evaluation code and usage examples provided.

### Dataset Format and Structure

Each problem is stored as a JSON object, with the following standardized fields:

- **problem_number**: An integer uniquely identifying each problem.
- **label**: A string categorizing the level of the problem.
- **problem_statement**: A string containing the LaTeX-formatted text of the mathematical problem.
- **answer**: A string providing the solution to the problem, formatted in LaTeX.
- **reasoning**: A string detailing the step-by-step solution or explanation, formatted in LaTeX.

An example of the data is as follows:

**problem_number:** 111

**label:** Olympiad-level

**problem_statement:** Let $S=\left\{1,2,\cdots \,,2024\right\}$. If any set of *n* pairwise relatively prime numbers in *S* has at least one prime number, the minimum value of *n* is —.

**answer:** 16

**reasoning:** Taking the 15 numbers 1, 2^2^, 3^2^, …, 43^2^ violates the condition. Furthermore, since *S* does not contain any non-prime numbers with a minimum prime factor of at least 47, there are only 14 types of non-prime numbers in *S*, excluding 1. Applying the Pigeonhole Principle, we conclude that *n* = 16.

### Dataset Overview and Composition

**Fig. 1:**
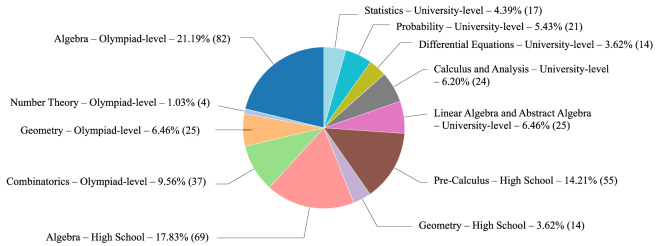
Mathematical problems across educational levels. We curate and categorize problems by difficulty and subject area.

The dataset contains 387 problems, evenly distributed across levels of difficulty and subject areas, as summarized in Fig. 1. Below we describe the key characteristics of the dataset.

#### Distribution by Difficulty Level

The dataset includes problems across three levels of increasing complexity: High School, University, and Olympiad. As shown in Table 3, the distribution is relatively balanced between High School (35.7%) and University (26.1%) levels, with a smaller but substantial portion (38.2%) allocated to Olympiad-level problems. This structure reflects the relative availability and design complexity of each category. The Olympiad-level problems were fewer in number due to their high construction and validation demands but are critical for testing advanced reasoning capabilities in LLMs.

**Table 3:** Distribution of problems by difficulty level.

Difficulty Level	Number of Problems	Percentage
High School	138	35.7%
University	101	26.1%
Olympiad-level	148	38.2%
Total	387	100%

#### Distribution by Answer Type

Each problem in the dataset is labeled with one of three answer types: Multiple Choice (MCQ), True/False (T-F), or Open-Ended (Open). As shown in Table 4, the majority of questions are in multiple-choice format (32.6%), which facilitates structured evaluation and model comparison. Open-ended questions account for 63.3% of the dataset and are designed to assess generative reasoning and mathematical expressiveness without reliance on fixed options. True/False questions, though fewer (4.1%), offer quick validation of specific claims and support binary classification tasks. This distribution reflects a deliberate balance between ease of evaluation and depth of reasoning assessment.

**Table 4:** Distribution of problems by answer type.

Answer Type	Number of Problems	Percentage
Multiple Choice (MCQ)	126	32.6%
True/False (T-F)	16	4.1%
Open-Ended (Open)	245	63.3%
Total	387	100%

#### Reasoning Annotations

Each problem includes a canonical reasoning trace that explains the steps leading to the final answer. These annotations support explainability and interpretability in model evaluation and may also serve as supervision signals for training or prompting LLMs.

### Software Package

A Python-based toolkit is included to streamline data analysis, offering functions for reading and analyzing data from the JSON files, along with tools for evaluating machine learning models. This toolkit is designed to assist researchers in efficiently handling the mathematical dataset.

## Technical Validation

The goal of the MathOdyssey dataset is to support rigorous, transparent, and reproducible evaluation of large language models (LLMs) in mathematical reasoning. To validate its structure and difficulty coverage, we conducted a series of controlled evaluations using representative LLMs. These experiments demonstrate the dataset’s ability to discriminate between models of different capability and highlight current challenges in math problem solving.

### Evaluation Protocol

A key advantage of the MathOdyssey data is that every question has an objective answer, so that it is straightforward to check the correctness by code. Such objective answers avoid subjective judgments from humans, making the evaluation consistent and reliable.

We use GPT-4 to assist in evaluating model accuracy, particularly for open-answer questions. The metric measures the similarity between the predicted and ground truth answers. In the MathOdyssey dataset, various types of questions and answers are included. We employ a prompt-based method to provide scores for evaluation, considering the following criteria:

- Mathematical Equivalence: Verify answers based on mathematical equivalence using advanced tools like symbolic computation software to confirm the equivalence of different algebraic or symbolic expressions.
- Scoring: Assign a score of ‘1’ for answers that match or are equivalent to the provided solution (exact value, choice label, or correctly rounded numerical approximation). Assign a score of ‘0’ for incorrect answers without providing explanatory feedback.
- Handling Multiple Choices: Consider the answer correct if the student correctly identifies the choice that matches the solution. Also, treat the corresponding choice as correct if the student provides the exact value that aligns with the problem’s context.
- Numerical Equivalence: Accept numerical answers that are correct to at least two decimal places or more, depending on the required precision.
- Symbolic and Algebraic Identities: Recognize and accept equivalent algebraic forms as correct, such as standard mathematical identities.
- Trigonometric and Logarithmic Forms: Accept equivalent trigonometric and logarithmic expressions, acknowledging transformations that change the form but not the value.
- Comprehensive Evaluation: Encourage the use of computational tools for checking equivalence in cases where expressions are too complex for straightforward visual inspection.

See the Supplementary Information for the evaluation prompts used in our method. The full evaluation code is publicly available, accompanied by comprehensive documentation and usage examples to support reproducibility and ease of use.

### Demonstrative Use: Evaluation of LLMs

To illustrate the dataset’s utility, we evaluated a range of contemporary LLMs using chain-of-thought prompting^6^. The models tested include GPT-4 o1-preview, GPT-4 Turbo, GPT-4, GPT-3.5 Turbo, Gemini models, Claude 3, and Llama-3-70B. All models are tested using chain-of-thought reasoning^6^. See the Supplementary Information for detailed descriptions of the problem-solving prompts.

We report the performance on our mathematical benchmarks, as shown in Table 5. Our observations indicate that the benchmark is challenging for these models, with overall performance below 60% except for GPT-4 o1-preview. (Advanced prompting methods using GPT-4 models in the contest can achieve performance improvements exceeding 60%.) The recent GPT-4 o1-preview achieves the highest overall performance at 65.12%, demonstrating that incorporating chain-of-thought learning significantly enhances capabilities. The Gemini Math-Specialized 1.5 Pro also performs well, ranking second with a score of 55.8%, suggesting that specialized training can further improve specific skill areas. GPT-4 Turbo achieves 49.35%, followed by Gemini 1.5 Pro at 45.0%, and Claude 3 Opus at 40.6%, all showing competitive performance. For closed-source models (specifically the GPT series) and state-of-the-art open-source models such as Llama-3, the results show that the selected open-source models not only surpass the performance of GPT-3.5 but are also approaching the capabilities of earlier versions of GPT-4.

**Table 5:** Results for different LLMs.

Model	Olympiad-level	High School	University-Level	Overall
GPT-4 o1-preview	45.27%	79.71%	74.26%	65.12%
GPT-4 Turbo	10.81%	84.06%	58.42%	49.35%
GPT-4	5.41%	85.51%	44.55%	44.19%
GPT-3.5 Turbo	3.38%	39.13%	16.83%	19.64%
Gemini				
- 1.5 Pro	—	—	—	45.0 %
- Math-Specialized 1.5 Pro	—	—	—	55.8 %
Claude 3 Opus	—	—	—	40.6 %
Llama-3-70B	8.78%	73.19%	24.75%	35.92%

The performance of Gemini 1.5 Pro and Claude 3 Opus are quoted from the Gemini 1.5 report^3^. Both GPT-4-Turbo and Gemini 1.5 Pro outperform the other models. For GPT-4-Turbo, we use results based on gpt-4-turbo-2024-04-09. For GPT-4, we use results based on gpt-4-0125. For GPT-3.5 Turbo, we use results based on gpt-3.5-turbo-0125.

When comparing different levels of mathematical problems for GPT models, we observe that High School mathematics is the easiest category for all models, with GPT-4 models scoring above 70%. Olympiad-level problems are the most difficult, with all models scoring below 11% except for GPT-4 o1-preview. Similar trends are seen for Llama-3-70B, with their performance in the Olympiad-level category being even lower, at less than 9%.

Furthermore, closed-source models, particularly the GPT-4 o1-preview and GPT-4 Turbo, exhibit stronger performance in high school and university-level math, highlighting ongoing advancements in their development. This data underscores the rapid progression of closed-source models in handling increasingly difficult mathematical questions over time. The performance gap between the previous closed-source model, GPT-4 Turbo, and the open-source Llama-3 for difficult mathematical problems is notably narrow. However, the gap between recent closed-source model GPT-4 o1-preview becomes larger. For instance, except that GPT-4 o1-preview achieves 45.27%, GPT-4 Turbo achieves an overall accuracy of 10.81% in the Olympiad-level mathematics, while Llama-3 achieves 8.78%. This demonstrates that both models, despite notable progress, still face significant challenges in solving these complex problems. However, for other difficulty levels, the gap becomes larger. For example, GPT-4 Turbo achieves 84.06% in high school mathematics, while Llama-3-70B scores only 73.19%, a difference of more than 10%.

## Limitations

The MathOdyssey dataset presents a diverse set of mathematical problems across multiple difficulty levels and subject areas. However, it may not capture the full breadth of mathematical reasoning strategies or problem-solving paradigms found in real-world or highly specialized contexts. As such, the generalizability of evaluation results to all types of mathematical reasoning tasks may be limited.

Additionally, while the current evaluation framework emphasizes objective correctness through comparison with canonical answers, it does not explicitly assess the quality of intermediate reasoning, creativity, or alternative solution paths. Future work may incorporate rubric-based human evaluation or structured analysis of step-by-step reasoning to address these aspects. Nonetheless, the current version of MathOdyssey provides a robust foundation for consistent, scalable benchmarking of mathematical reasoning in large language models.

## Usage Notes

The MathOdyssey dataset comprises a range of problems from Olympiad-level competitions, advanced high school curricula, and university-level mathematics, created by mathematics professionals, including high school educators, researchers, and university professors. We are pleased to distribute the dataset under the CC BY-SA 4.0” license.

There are no access restrictions or no limitations on data use for our collected dataset.

Please see the GitHub repository mentioned in the next section for example scripts regarding baseline models and evaluation.

## Supplementary information

Supplementary Information: MathOdyssey

## Data Availability

The code is available in a GitHub repository at https://github.com/MathOdyssey/odyssey-math. The “jsonl” folder contains the results of the baseline models.
